# LncRNA BCRT1 facilitates osteosarcoma progression via regulating miR-1303/FGF7 axis

**DOI:** 10.18632/aging.203106

**Published:** 2021-06-08

**Authors:** Gang Han, Quanyi Guo, Ning Ma, Wenzhi Bi, Meng Xu, Jinpeng Jia, Wei Wang

**Affiliations:** 1 Department of Orthopedics, The First Medical Center of General Hospital of PLA, Beijing 100853, China

**Keywords:** osteosarcoma, FGF7, miR-1303, BCRT1

## Abstract

Growing studies noted that lncRNA was closely related with the initiation and progression of tumors. However, the role of BCRT1 in the progression of osteosarcoma remains unknown. We noted that BCRT1 is significantly upregulated in osteosarcoma specimens and cells. Elevated expression of BCRT1 promotes cell growth and cell cycle in osteosarcoma cell. Moreover, BCRT1 induces EMT and secretion of inflammatory mediators in osteosarcoma cell. We illustrated that elevated expression of BCRT1 decreases miR-1303 expression in MG-63 cell. The expression of miR-1303 is lower in osteosarcoma specimens than in non-tumor specimens. There is an inverse interrelation between miR-1303 levels and BCRT1 levels in osteosarcoma specimens. Furthermore, we identified FGF7 is one direct target gene of miR-1303 in osteosarcoma cell. Ectopic expression of miR-1303 suppresses FGF7 expression and elevated expression of BCRT1 enhanced FGF7 expression in MG-63 cell. Finally, we illustrated that BCRT1 induces osteosarcoma cell cycle and proliferation and promotes EMT progression and inflammatory mediators secretion via modulating FGF7 expression. Our study suggested that BCRT1 acts as one oncogene in osteosarcoma progression.

## INTRODUCTION

Osteosarcoma is the 8^th^ leading tumor with approximately incidence of 4.4 per one million. Osteosarcoma mostly occurs in adolescents and children, contributing to about 5% of all childhood malignancies and about 9% of tumor-correlated deaths in children [1–4]. Clinical results illustrated that this cancer has an uncertain prognosis, even with comprehensive therapies including chemotherapy and amputation [3, 5, 6]. Extensive cancer-associated specific mediators and signaling pathways have been identified in osteosarcoma prognosis, pathogenesis and progression [7–10]. However, the molecular mechanisms about these specific mediators and signaling pathways are still largely unknown in osteosarcoma carcinogenesis. Thus, it is important to study molecular mechanisms about osteosarcoma pathogenesis.

LncRNAs are one subset of RNAs with length of > 200 nucleotides and no protein-coding abilities [11–17]. LncRNAs were discovered to deregulate expressed in several tumors including in osteosarcoma [18–21]. Growing studies have proved that lncRNAs participate in pathological and physiological processes such as cell development, metabolism, apoptosis, cycle and invasion [11, 18, 22–27]. Recently, Liang et al [28]. noted that BCRT1 is overexpressed in breast tumor tissues and is associated with poor prognosis. Knockdown of BCRT1 inhibits tumor metastasis and growth. However, the role of BCRT1 in progression of osteosarcoma remains unknown.

## RESULTS

### BCRT1 is significantly upregulated in osteosarcoma specimens

**Figure 1:**
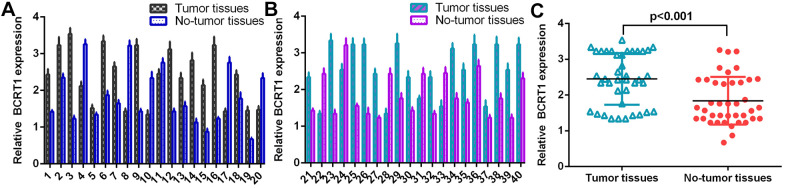
**BCRT1 was significantly upregulated in osteosarcoma specimens.** (**A**) The level of BCRT1 in osteosarcoma case 1-20 was measured by RT-qPCR assay. (**B**) The expression of BCRT1 in osteosarcoma case 21-40 was determined by RT-qPCR analysis. (**C**) The expression of BCRT1 was higher in osteosarcoma specimens than in no-tumor specimens.

The level of BCRT1 was determined in 40 osteosarcoma specimens using RT-qPCR analysis and data were shown in Figure 1A, 1B. The expression of BCRT1 is higher in osteosarcoma specimens than in non-tumor specimens (Figure 1C).

### MiR-1303 is significantly downregulated in osteosarcoma specimens

**Figure 2:**
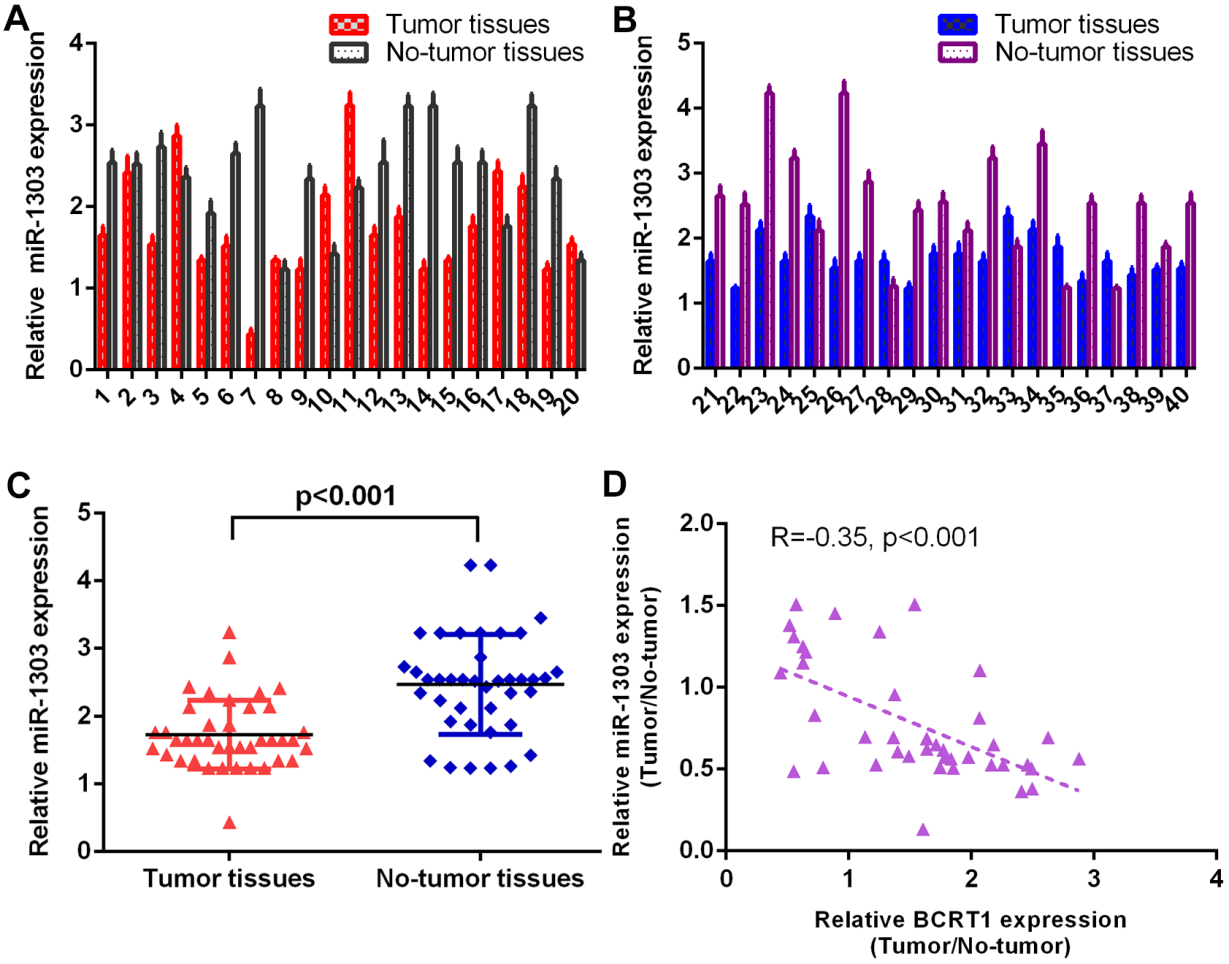
**miR-1303 was significantly downregulated in osteosarcoma specimens.** (**A**) The level of miR-1303 in osteosarcoma case 1-20 was measured by RT-qPCR assay. (**B**) The expression of miR-1303 in osteosarcoma case 21-40 was determined using RT-qPCR analysis. (**C**) The expression of miR-1303 was lower in osteosarcoma specimens than in no-tumor specimens. (**D**) The expression of miR-1303 was inversely interrelated with BCRT1 in osteosarcoma specimens.

The level of miR-1303 was measured in 40 osteosarcoma specimens using RT-qPCR method and data were shown in Figure 2A, 2B. The expression of miR-1303 is lower in osteosarcoma specimens than in non-tumor specimens (Figure 2C). As indicated in Figure 2D, Spearman’s correlation assay shows an inverse interrelation between miR-1303 levels and BCRT1 levels.

### BCRT1 enhances osteosarcoma cell cycle and proliferation

**Figure 3:**
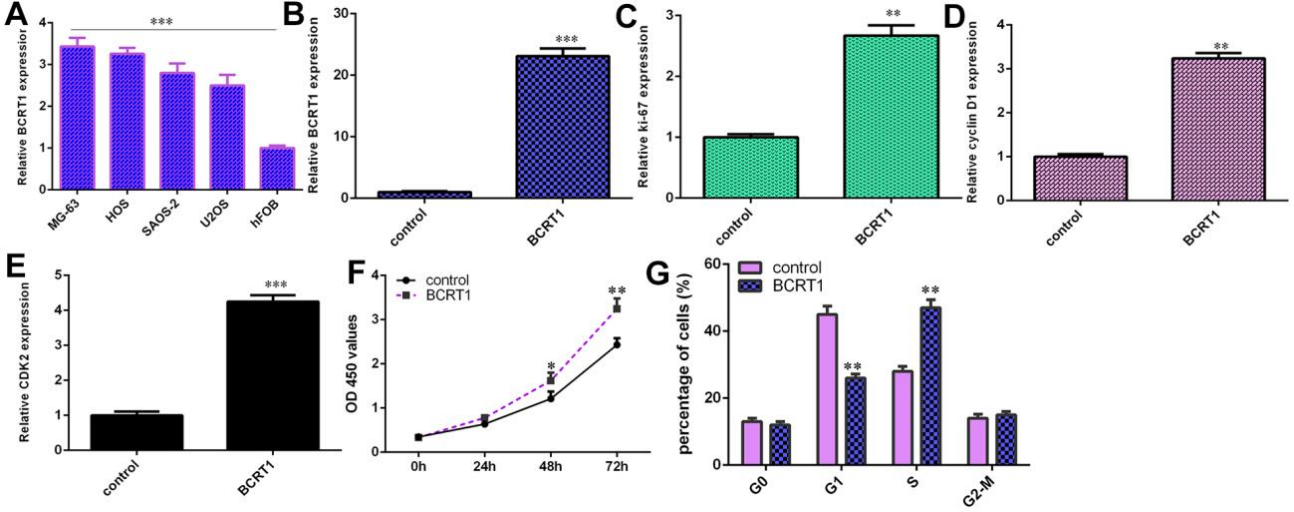
**BCRT1 enhanced osteosarcoma cell cycle and proliferation.** (**A**) The expression of BCRT1 in four osteosarcoma cell lines (MG-63, HOS, SAOS-2 and U2OS) and hFOB was determined by RT-qPCR analysis. (**B**) The level of BCRT1 was detected using RT-qPCR analysis. (**C**) Ectopic expression of BCRT1 increased ki-67 expression in MG-63 cell. (**D**) The level of cyclin D1 was determined using RT-qPCR method. (**E**) The level of CKD2 was determined using RT-qPCR method. (**F**) Elevated expression of BCRT1 promoted cell growth in MG-63 cell. (**G**) Overexpression of BCRT1 increased cell cycle in MG-63 cell. *p<0.05, **p<0.01 and ***p<0.001.

RT-qPCR assay illustrated higher level of BCRT1 in four osteosarcoma cell lines (MG-63, HOS, SAOS-2 and U2OS) compared to hFOB (Figure 3A). Increased expression of BCRT1 is identified in MG-63 cell after treatment with pcDNA- BCRT1 using qRT-PCR (Figure 3B). Ectopic expression of BCRT1 increases the expression ki-67 (Figure 3C), cyclin D1 (Figure 3D) and CKD2 (Figure 3E) in MG-63 cell. Elevated expression of BCRT1 promotes cell growth in MG-63 cell (Figure 3F). Overexpression of BCRT1 increases cell cycle in MG-63 cell (Figure 3G).

### BCRT1 induces EMT progression and secretion of inflammatory mediators in osteosarcoma cell

**Figure 4:**
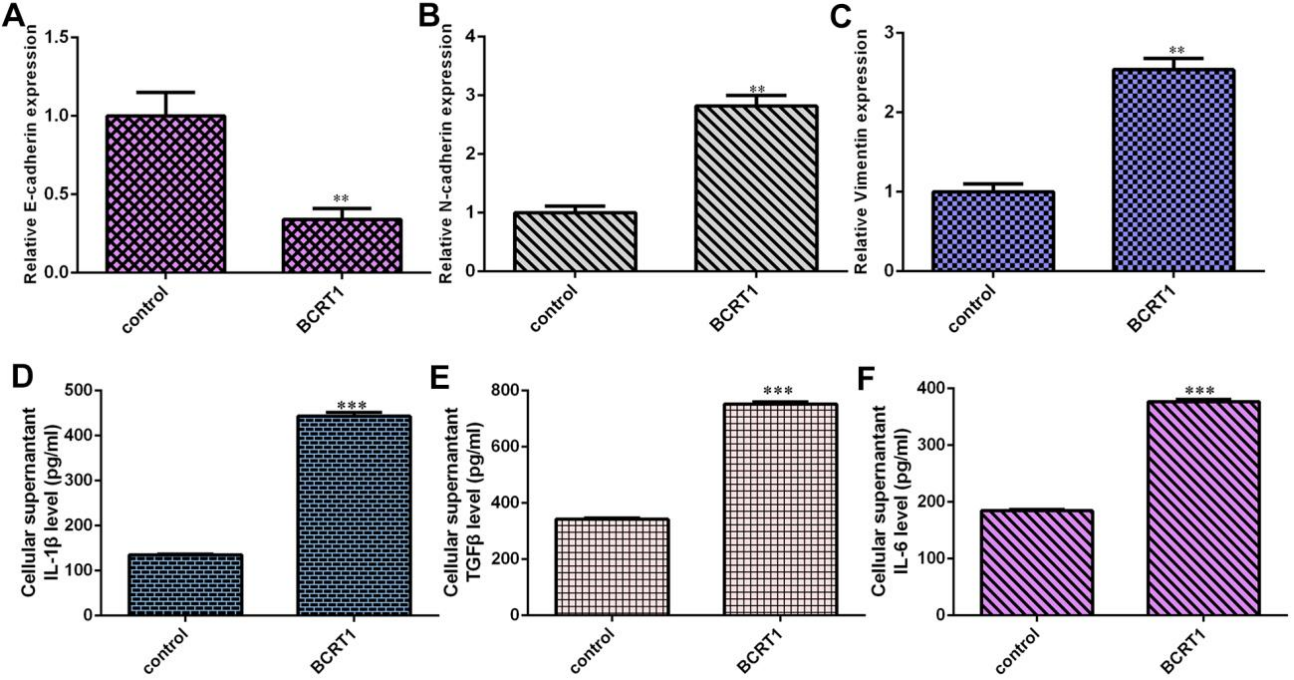
**BCRT1 induced EMT progression and inflammatory mediators secretion in osteosarcoma cell.** (**A**) Overexpression of BCRT1 inhibited E-cadherin expression in MG-63 cell. (**B**) The expression of N-cadherin was detected by qRT-PCR assay. (**C**) The level of vimentin was measured using qRT-PCR assay. (**D**) Elevated expression of BCRT1 induced IL-1β secretion. (**E**) The level of TGFβ was determined by ELISA assay. (**F**) The level of IL-6 was measured by ELISA assay. **p<0.01 and ***p<0.001.

Overexpression of BCRT1 inhibits epithelial marker E-cadherin expression in MG-63 cell (Figure 4A). Moreover, elevated expression of BCRT1 increases the expression of N-cadherin (Figure 4B) and vimentin (Figure 4C) in MG-63 cell, which were the mesenchymal markers. Elevated expression of BCRT1 induces secretion of several inflammatory mediators, including IL-1β (Figure 4D), TGFβ (Figure 4E) and IL-6 (Figure 4F).

### BCRT1 regulates miR-1303/FGF7 expression in osteosarcoma cell

**Figure 5:**
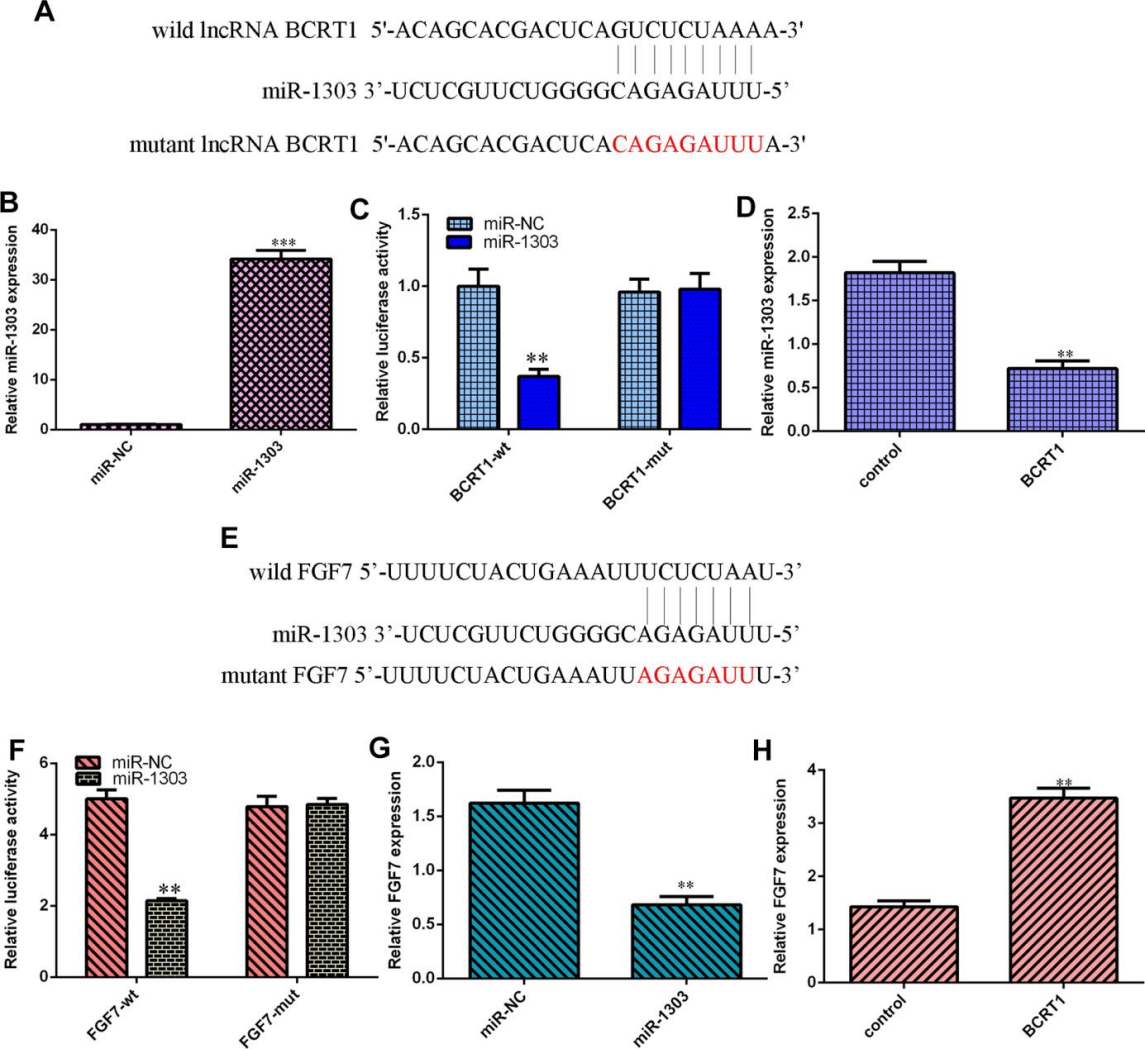
**BCRT1 regulated miR-1303/FGF7 expression in osteosarcoma cell.** (**A**) BCRT1 may sponge miR-1303 expression using starbase bioinformatics algorithm. (**B**) The expression of miR-1303 was determined by qRT-PCR assay. (**C**) miR-1303 overexpression suppressed luciferase value of wild BCRT1 3’UTR but not the mutant BCRT1 3’UTR in MG-63 cell. (**D**) Elevated expression of BCRT1 decreased miR-1303 expression in MG-63 cell. (**E**) FGF7 may one target gene of miR-1303 using targetscan bioinformatics algorithm. (**F**) Luciferase reporter analysis noted that miR-1303 overexpression suppresses luciferase value of wild FGF7 3’UTR but not the mutant FGF7 3’UTR in MG-63 cell. (**G**) Ectopic expression of miR-1303 suppressed FGF7 expression in MG-63 cell. (**H**) Elevated expression of BCRT1 enhanced FGF7 expression in MG-63 cell. **p<0.01 and ***p<0.001.

Our analysis indicated that BCRT1 might sponge miR-1303 expression using starbase bioinformatics algorithm (Figure 5A). Increased expression of miR-1303 is identified in MG-63 cell after treatment with miR-1303 mimic using qRT-PCR (Figure 5B). Luciferase reporter analysis noted that miR-1303 overexpression suppresses luciferase value of wild BCRT1 3’UTR but not the mutant BCRT1 3’UTR in MG-63 cell (Figure 5C). Elevated expression of BCRT1 decreases miR-1303 expression in MG-63 cell (Figure 5D). Next, our analysis noted that FGF7 may be one target gene of miR-1303 using targetscan bioinformatics algorithm (Figure 5E). Luciferase reporter analysis noted that miR-1303 overexpression suppresses luciferase value of wild FGF7 3’UTR but not the mutant FGF7 3’UTR in MG-63 cell (Figure 5F). Ectopic expression of miR-1303 suppresses FGF7 expression in MG-63 cell (Figure 5G). Elevated expression of BCRT1 enhances FGF7 expression in MG-63 cell (Figure 5H).

### BCRT1 induces osteosarcoma cell cycle and proliferation via modulating FGF7 expression

**Figure 6:**
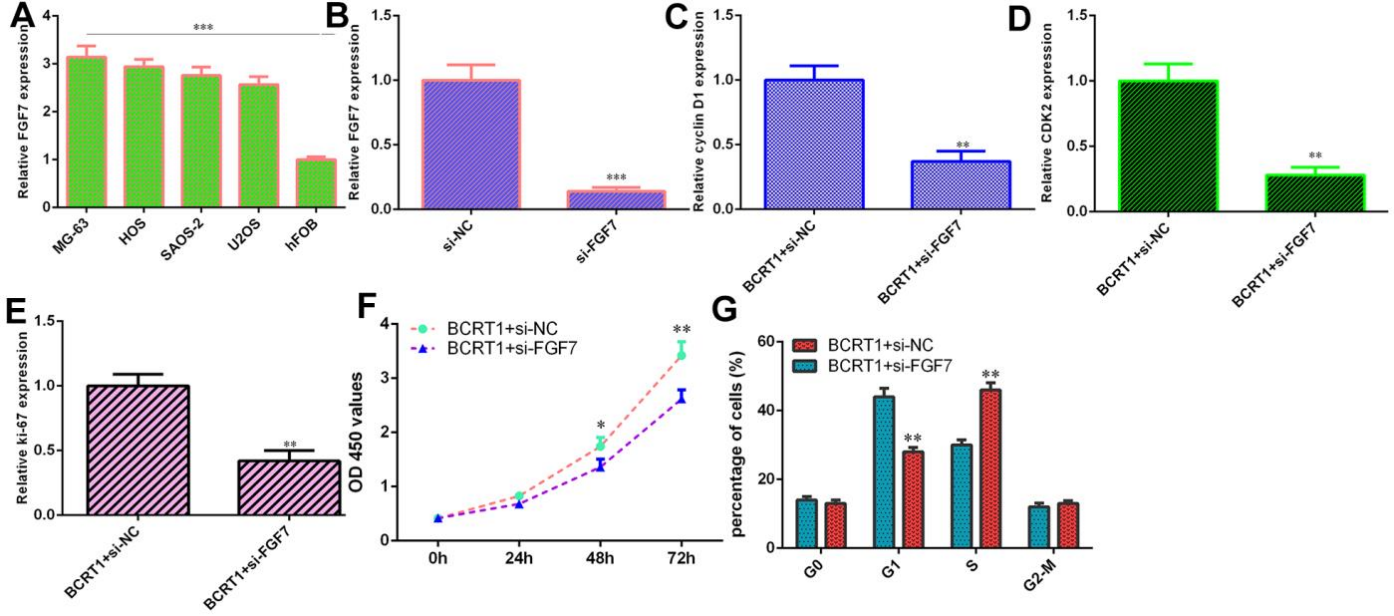
**BCRT1 induced osteosarcoma cell cycle and proliferation via modulating FGF7 expression.** (**A**) The level of FGF7 in four osteosarcoma cell lines (MG-63, HOS, SAOS-2 and U2OS) and hFOB was detected using qRT-PCR assay. (**B**) The expression of FGF7 was determined by qRT-PCR assay. (**C**) The expression of cyclin D1 was measured using qRT-PCR assay. (**D**) The expression of CKD2 was measured using qRT-PCR assay. (**E**) The level of ki-67 was detected using qRT-PCR assay. (**F**) Cell proliferation was measured by CCK-8 assay. (**G**) Inhibition expression of FGF7 suppressed cell cycle in BCRT1-overexpressing MG-63 cell. *p<0.05, **p<0.01 and ***p<0.001.

RT-qPCR assay illustrates higher level of FGF7 in four osteosarcoma cell lines (MG-63, HOS, SAOS-2 and U2OS) compared to hFOB (Figure 6A). Decreased expression of FGF7 is identified in MG-63 cell after treatment with FGF7 siRNA using qRT-PCR (Figure 6B). Knockdown of FGF7 decreases cyclin D1 (Figure 6C), CKD2 (Figure 6D) and ki-67 (Figure 6E) in BCRT1-overexpressing MG-63 cell. Inhibited expression of FGF7 suppresses cell growth (Figure 6F) and cell cycle (Figure 6G) in BCRT1-overexpressing MG-63 cell.

### BCRT1 promotes EMT progression and secretion of inflammatory mediators through regulating FGF7 expression

**Figure 7:**
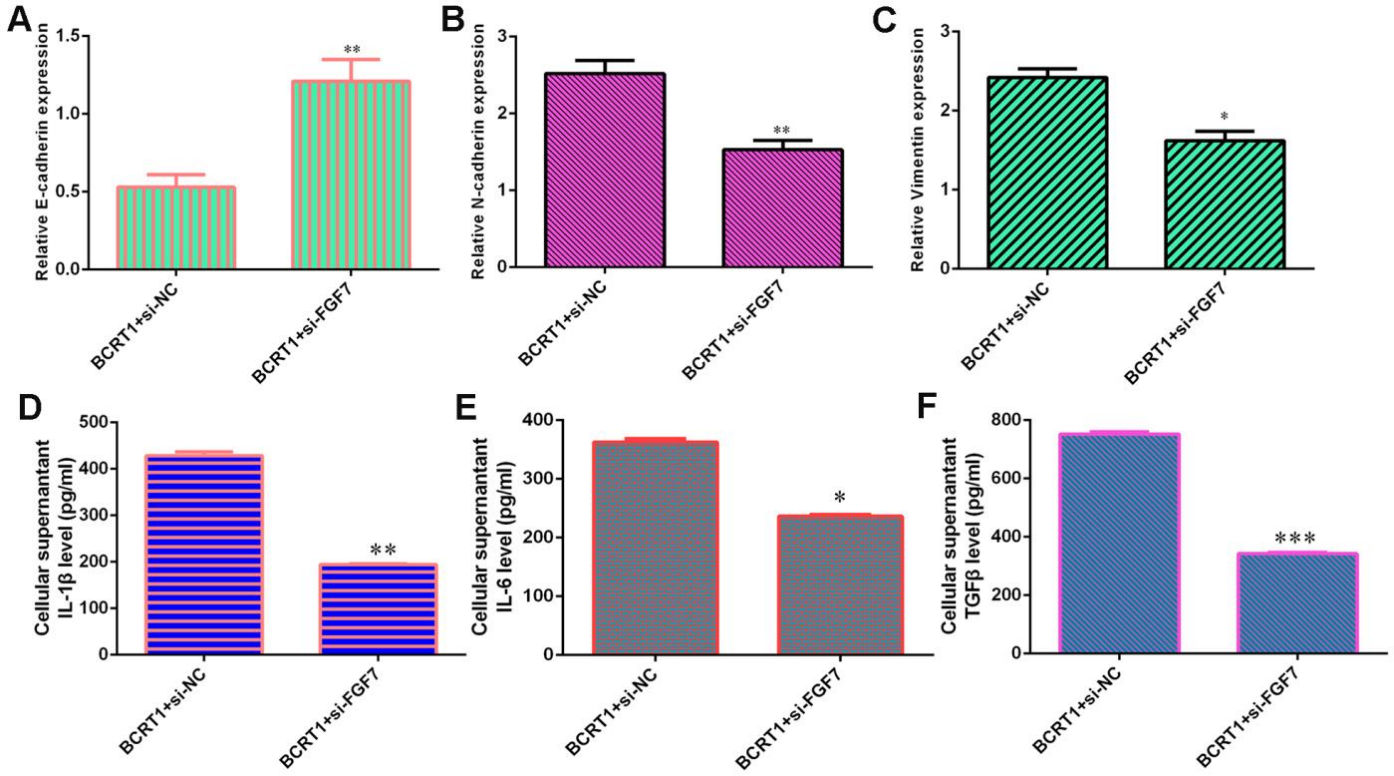
**BCRT1 promoted EMT progression and inflammatory mediators secretion through regulating FGF7 expression.** (**A**) The level of E-cadherin was measured by qRT-PCR assay. (**B**) The expression of N-cadherin was determined with qRT-PCR assay. (**C**) The expression of vimentin was studied with qRT-PCR analysis. (**D**) The level of IL-1β was determined using ELISA assay. (**E**) The level of IL-6 was measured by ELISA assay. (**F**) The level of TGFβ was determined using ELISA assay. *p<0.05, **p<0.01 and ***p<0.001.

Knockdown of FGF7 promotes E-cadherin expression (Figure 7A) and inhibits N-cadherin (Figure 7B) and vimentin (Figure 7C) expression in BCRT1-overexpressing MG-63 cell. Furthermore, inhibited expression of FGF7 decreases secretion of inflammatory mediators including IL-1β (Figure 7D), IL-6 (Figure 7E) and TGFβ (Figure 7F) in BCRT1-overexpressing MG-63 cell.

### FGF7 induces osteosarcoma cell cycle and proliferation, EMT progression and secretion of inflammatory mediators

**Figure 8:**
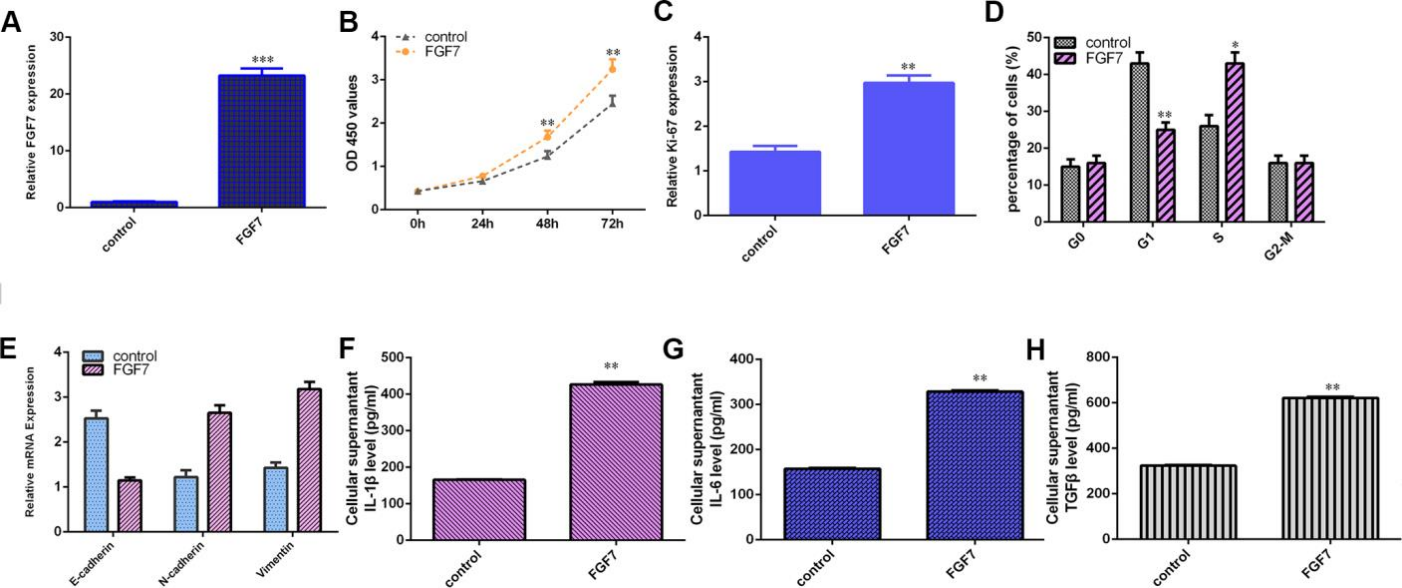
**FGF7 induces osteosarcoma cell cycle and proliferation, EMT progression and secretion of inflammatory mediators.** (**A**) The expression of FGF7 was measured by qRT-PCR assay. (**B**) Ectopic expression of FGF7 increased cell growth in the MG-63 cell. (**C**) The expression of ki-67 was measured by qRT-PCR assay. (**D**) Overexpression of FGF7 increased cell cycle in MG-63 cell. (**E**) Elevated expression of FGF7 suppressed E-cadherin expression and increased the expression of N-cadherin and vimentin in MG-63 cell. (**F**) The level of IL-1β was determined by ELISA assay. (**G**) The level of IL-6 was determined by ELISA assay. (**H**) The level of TGFβ was determined by ELISA assay. *p<0.05, **p<0.01 and ***p<0.001.

The level of FGF7 was overexpressed in MG-63 cell after treatment with pcDNA-FGF7 by qRT-PCR (Figure 8A). Ectopic expression of FGF7 increased cell growth (Figure 8B) and ki-67 (Figure 8C) expression in the MG-63 cell. Overexpression of FGF7 increased cell cycle in MG-63 cell (Figure 8E). Elevated expression of FGF7 suppressed E-cadherin expression and increased the expression of N-cadherin and vimentin in MG-63 cell (Figure 8F). Ectopic expression of FGF7 induces secretion of several inflammatory mediators, including IL-1β (Figure 8F), IL-6 (Figure 8G) and TGFβ (Figure 8H).

## DISCUSSION

Recent studies have illustrated that lncRNAs participate in the progression of various tumors, including osteosarcoma. For instance, Liu et al [29]. noted that PGM5-AS1 induces EMT progression, metastasis and invasion of osteosarcoma cells through impairing miR-140-5p-regulated FBN1 inhibition. Liu et al [30]. showed that FOSL2 and OIP5-AS1 is overexpressed in cisplatin resistant osteosarcoma cells and tissues. Knockdown of OIP5-AS1 inhibits osteosarcoma growth and induces cisplatin sensitivity of osteosarcoma. Shen et al [31]. indicated that lncARSR confers resistance to drug Adriamycin and induced progression of osteosarcoma. Furthermore, Liang et al [28]. noted that BCRT1 is overexpressed and is associated with poor prognosis in breast tumor. Knockdown of BCRT1 inhibits tumor metastasis and growth. However, the role of BCRT1 in the progression of osteosarcoma remains unknown. In present research, we noted that BCRT1 is significantly upregulated in osteosarcoma specimens and cells. Elevated expression of BCRT1 promotes cell growth and cell cycle in osteosarcoma cell. We also noted that overexpression BCRT1 increased ki-67, cyclin D1 and CKD2 expression due to these genes were the important proliferation markers. Moreover, BCRT1 induces EMT progression and secretion of inflammatory mediators in osteosarcoma cell.

Growing literatures have illustrated that lncRNAs serve as miRNA sponges or decoys to influence their expression. For example, Wang et al [32]. found that RHPN1-AS1 acts as an oncogene in osteosarcoma via sponging miR-506/SNAI2 Expression. LncRNA LINC01419 was noted to regulate PDRG1/miR-519a-3p axis to induced osteosarcoma cell progression. Chen et al [33]. illustrated that knockdown of LINC00313 suppresses metastasis and tumorigenesis in osteosarcoma via modulating miR-342-3p/FOSL2 axis. Zhang et al [34]. showed that DSCAM-AS1 induces USP47 expression via sponging miR-101-3p to promote progression of osteosarcoma. Wang et al [35]. noted that lncRNA OR3A4 modulated osteosarcoma cells growth through regulating miR-1207-5p/G6PD axis. Recently, Liang et al [28]. showed that knockdown of BCRT1 inhibits tumor metastasis and growth through regulating miR-1303/PTBP3 axis. We also illustrated that elevated expression of BCRT1 decreases miR-1303 expression in MG-63 cell. The expression of miR-1303 is lower in osteosarcoma specimens than in non-tumor specimens. There is an inverse interrelation between miR-1303 level and BCRT1 level in osteosarcoma specimens. Furthermore, we identified FGF7 is one direct target gene of miR-1303 in osteosarcoma cell. Ectopic expression of miR-1303 suppresses FGF7 expression and elevated expression of BCRT1 enhances FGF7 expression in MG-63 cell. Finally, we illustrated that BCRT1 induces osteosarcoma cell cycle and proliferation and promotes EMT progression and inflammatory mediators secretion via modulating FGF7 expression. Overexpression of FGF7 induced osteosarcoma cell cycle and proliferation, EMT progression and secretion of inflammatory mediators.

Taken together, present study illustrated that BCRT1 is significantly upregulated in osteosarcoma specimens and BCRT1 induces osteosarcoma cell cycle and proliferation and promotes EMT progression and secretion of inflammatory mediators via modulating FGF7 expression. It suggested that BCRT1 acts as one oncogene in osteosarcoma progression.

## MATERIALS AND METHODS

### Tissue specimens and cell culture and transfection

Osteosarcoma specimens and adjacent specimens were collected from cases undergoing surgery at our department and all cases gave informed consent. The biopsies were kept in liquid nitrogen until RNA extraction. Osteosarcoma cells (U2OS, SAOS-2, MG-63 and HOS) and hFOB1.19 cell was cultured in the RPMI-1640 medium supplemented by penicillin and FBS. miR-NC mimic and miR-1303 mimic, pcDNA-BCRT1 and pcDNA-control, siRNA FGF7 and control vectors, pcDNA-FGF7 and pcDNA-control vectors were obtained from GenePharma Company (Shanghai, China). Cell transfection was carried out using Lipofectamin3000 (Invitrogen Inc.)

### RT-qPCR assay

Total RNAs from MG-63 cell and specimens were separated using TRIzol kit (Thermo Fisher, Inc.) following to standard protocol. RT-qPCR was carried out to detect expression level of miRNA, mRNA and lncRNA on 7900HT PCR system (Thermo Fisher, Inc.) using SYBR Green reagent. The primers were: BCRT1, F, 5-‘TCTCGCTTCTGAGTTGGTGAC-3’; R, 5-‘GAAAGCTCTGGCAGTGTTGGA-3’; miR-1303, F, 5-‘TTTAGAGACGGGGTCTTGCTCT-3’; R, 5-‘CAGTGCGTGTCGTGGAGT-3’; U6, F, 5-‘CTCGCTTCGGCAGCACA-3’; R, 5-‘AACGCTTCACGAATTTGCGT-3’. E-cadherin, F,5’-ATGCCATCGTTGTTCACTGGA-3’; R,5’-CATGAGAAGTATGACAACAGCCT-3’; N-cadherin, F, 5’-CCGGAGAACAGTCTCCAACTC-3’; R, 5’-CCCACAAAGAGCAGCAGTC-3’; vimentin, F, 5’-CCCTCACCTGTGAAGTGGAT-3’; R, 5’-TCCAGCAGCTTCCTGTAGGT-3’; Ki-67, F, 5’- TCCTTTGGTGGGCACCTAAGACCTG-3’; R, 5’- TGATGGTTGAGGTCGTTCCTTGATG-3’;Cyclin D1 F, 5’- AAC TACCTGGACCGCTTCCT-3’; R, 5’- CCACTT GAGCTTGTTCACCA The relative level of miRNA was normalized to U6 and mRNA and lncRNA was normalized to GAPDH. The expression was determined using method of 2^−ΔΔCq^.

### Cell growth and cell cycle and colony formation

The viability of osteosarcoma cell cultured in the 96-well dish was detected by CCK-8 analysis reagent (Dojindo, Japan). After treatment, 10 μL CCK-8 kit was added into each well for 3 hours. Absorbance at the 450 nm was read at plate reader. Cell stages of cycle were evaluated using flow cytometry. Cells were fixed in the ethanol (70%, ice) and treated with 10 mg/mL RNase A. Cell was stained with propidium iodide and were analyzed by FACStar sorter. For cell colony formation, cells were cultured in the 96-well and cells continued to culture for 7 days. Colonies were stained with the crystal violet and pictured.

### Dual-luciferase reporter gene assay

The mut and WT reporter vectors of BCRT1 and FGF7 (mut- BCRT1 or FGF7 and WT- BCRT1 or FGF7) were synthetized from GenePharma Company (Shanghai, China). These NC mimics and miR-1303 mimics were co-transfected with the mut- BCRT1 or FGF7 and WT- BCRT1 or FGF7 into MG-63 cell. After 48 hours luciferase values were studied at luciferase reporter kit (Promega Corporation, USA) following to protocol.

### Enzyme-linked immunosorbent assay (ELISA)

Level of IL-1β, IL-6 and TGFβ in cell culture supernatant was detected with ELISA using IL-1β, IL-6 and TGFβ ELISA reagent (R&D Systems).

### Statistical analysis

All data were noted as mean ± SD (standard deviation). Student’s t-test was utilized to compare the difference of 2 groups. P value with less than 0.05 was indicated as significant difference.
